# A Strategy for Comparing the Contributions of Environmental Chemicals and Other Risk Factors to Neurodevelopment of Children

**DOI:** 10.1289/ehp.1104170

**Published:** 2011-12-19

**Authors:** David C. Bellinger

**Affiliations:** Children’s Hospital Boston, Harvard Medical School, Boston, Massachusetts, USA

**Keywords:** chemicals, children, epidemiology, neurodevelopment

## Abstract

Background: The impact of environmental chemicals on children’s neurodevelopment is sometimes dismissed as unimportant because the magnitude of the impairments are considered to be clinically insignificant. Such a judgment reflects a failure to distinguish between individual and population risk. The population impact of a risk factor depends on both its effect size and its distribution (or incidence/prevalence).

Objective: The objective was to develop a strategy for taking into account the distribution (or incidence/prevalence) of a risk factor, as well as its effect size, in order to estimate its population impact on neurodevelopment of children.

Methods: The total numbers of Full-Scale IQ points lost among U.S. children 0–5 years of age were estimated for chemicals (methylmercury, organophosphate pesticides, lead) and a variety of medical conditions and events (e.g., preterm birth, traumatic brain injury, brain tumors, congenital heart disease).

Discussion: Although the data required for the analysis were available for only three environmental chemicals (methylmercury, organophosphate pesticides, lead), the results suggest that their contributions to neurodevelopmental morbidity are substantial, exceeding those of many nonchemical risk factors.

Conclusion: A method for comparing the relative contributions of different risk factors provides a rational basis for establishing priorities for reducing neurodevelopmental morbidity in children.

Assessments of the import of an association observed between an environmental chemical exposure and child neurodevelopment often focus solely on the magnitude of the effect size and its associated *p*-value (i.e., whether it is < 0.05). Effect size is expressed in various forms, as the difference between the mean scores of exposed and unexposed groups, the change in score per unit change in an exposure biomarker, or the change in risk (relative risk, odds ratio) associated with a particular value of the biomarker. Among the reasons cited to dismiss an effect size is that it is clinically unimportant (e.g., Kaufman 2001). This perspective fails to place the effect estimate in a public health context, however. Estimating the population burden attributable to a factor requires a metric that reflects not only the magnitude of the risk associated with the factor but also the frequency with which the factor occurs in the population (Steenland and Armstrong 2006), a concept embodied in the environmentally attributable fraction model (Institute of Medicine 1981). Although a factor associated with a large impact would be a significant burden to a patient, it might not be a major contributor to population burden if it occurs rarely. Conversely, a factor associated with a modest but frequently occurring impact could contribute substantially to population burden.

The objective of this review was to describe a population-oriented approach to estimating risk factor burden as an alternative to the usual disease-oriented approach. This approach was then used to compare the population burdens associated with major medical, social, and chemical risks to child development.

## Methods

Several choices were required to apply the approach.

*Risk factors.* An effort was made to estimate the contributions to children’s neurodevelopment of a wide range of medical conditions, including neurodevelopmental disorders, postnatal events, socioeconomic and psychosocial risks. The goal was not to provide an exhaustive accounting of all contributors, however, and the selection of risk factors was not based on a systematic review, but on the availability of data.

*End point for comparison.* In most studies, a battery of neurodevelopmental tests assessing many domains is administered. Full-Scale IQ (FSIQ) score is the end point most consistently reported across studies, however, and provides the best opportunity to conduct a comparative analysis. Among the instruments used to assess neurodevelopment in children, FSIQ tests tend to be the strongest psychometrically, and an extensive body of research demonstrates relationships between FSIQ and many important late outcomes (Neisser et al. 1996).

*Evidence on which effect sizes are based.* Given the typical heterogeneity of the effect sizes derived from individual epidemiologic studies, basing the calculation on the effect size from a single study, particularly one of the first reported (Ioannidis 2005), would be an invitation to controversy. An effect estimate derived from a meta-analysis or pooled analysis, conducted by subject matter experts applying a transparent protocol for study inclusion and weighting, reflects the weight of evidence available on a risk factor. Therefore, preference was given to effect estimates based on a meta-analysis or pooled analysis that provided evidence supporting a statistically significant association between a risk factor and FSIQ. In some cases, when such an analysis was not available, a single study was used, preferably one that was population based and with a large sample size. However, this was done in recognition that the effect size would likely differ, to an uncertain extent, if based on an analysis that integrated the findings of multiple studies.

*Population to which to generalize findings.* A population of 25.5 million 0- to 5-year-old children in the United States was assumed (Federal Interagency Forum on Child and Family Statistics 2011).

*Method used to estimate FSIQ loss.* For a categorical risk factor (i.e., one that a child either has or does not), the published prevalence of the factor in 0- to 5-year-old children was multiplied by the difference between the FSIQ scores of children with and without the risk factor (i.e., the effect size) to estimate the total FSIQ loss associated with it. If only an estimate of yearly incidence was available (e.g., the number of events per year among children 0–5 years of age), the incidence was multiplied by five (the number of yearly intervals in a population of 0- to 5-year-olds) to estimate the total number of children in the cohort who would be expected to have experienced the risk factor. When the prevalence/incidence among 0- to 5-year-olds was not available, it was estimated based on the prevalence/incidence for the closest age range for which it was available.

For risk factors represented by a continuous distribution, the numbers of 0- to 5-year-old children (or the age range closest to this) with values in specified intervals of the distribution were calculated (e.g., 25th–50th percentile; 90th–95th percentile). Based on the dose–effect relationship for the risk factor, slopes for the FSIQ loss per unit increase in the factor were calculated, either across the entire distribution or, in the case of a nonlinear relationship, over specific intervals of the exposure distribution. Within each interval, the slope was multiplied, first, by the midpoint value of the risk factor (or the distance between the midpoint value and the value below which no association has been found to exist between the risk factor and FSIQ) and, second, by the estimated number of children in the interval. The FSIQ losses of children within intervals were then summed to derive the total number of FSIQ points lost. (See Table 1 for an example.)

**Table 1:** Estimated numbers of FSIQ points lost from lead exposure for children in different intervals of the blood lead distribution.

Range of the BLL distribution (µg/dL)	Midpoint BLL (µg/dL)	FSIQ loss*a*	No. of children	No. of FSIQ points lost
0 to 50th percentile (0–1.43)		0.72		0.37		12,750,000		4,717,500
50th to 75th percentile (1.43–2.10)		1.77		0.90		6,375,000		5,737,500
75th to 90th percentile (2.10–2.98)		2.54		1.30		3,825,000		4,972,500
90th to 95th percentile (2.98–3.80)		3.39		1.73		1,275,000		2,205,750
95th to 98th percentile (3.80–7.50)		5.65		2.88		765,000		2,203,200
> 98th percentile (> 7.50)		15		6.10		510,000		3,111,000
Abbreviation: BLL, blood lead level. **a**The FSIQ losses associated with blood lead intervals < 98th percentile were calculated by multiplying the midpoint BLL in an interval by –0.51 IQ points/µg/dL, the slope estimated for BLL < 10 µg/dL in the pooled analysis (Lanphear et al. 2005). The midpoint BLL of children with a BLL > 98th percentile was assumed to be 15, and the FSIQ loss was assumed to be 6.10 points (5.1 points lost up to a BLL of 10 µg/dL and an additional 1 point, which is one-half of the FSIQ loss estimated in the pooled analyses to be associated with an increase in BLL from 10 to 20 µg/dL).								

The level of precision in the calculations reported here should not be overinterpreted. They are intended to provide only rough estimates of the FSIQ losses associated with different risk factors for purposes of comparison.

## Results

*Medical conditions.* Congenital heart disease. Karsdorp et al. (2007) conducted a meta-analysis in which estimates were derived for the FSIQ deficits associated with different forms of congenital heart disease, the most common structural birth defect. The scores of children with a ventricular or atrial septal defect were not impaired, but the scores of children with more complex lesions—such as tetralogy of Fallot (TOF), d-transposition of the great arteries (d-TGA), and hypoplastic left heart syndrome (HLHS)—were, with mean deficits of 2.55, 2.10, and 12.3 points, respectively. The incidence of TOF is 4 in 10,000 (Child 2004), or 10,200 cases in children 0–5 years of age in the United States. The incidence of d-TGA is 3 in 10,000 (Martins and Castela 2008), or 7,650 cases, and the incidence of HLHS is 2 in 10,000 (Barron et al. 2009), or 5,100 cases. Therefore, the estimated numbers of FSIQ points lost are 26,010 (TOF), 16,065 (d-TGA), and 62,730 (HLHS), totaling 104,805 points.

Preterm birth. Approximately 12.3% of U.S. children are born preterm (gestational age < 37 weeks) (Hamilton et al. 2010). In a meta-analysis of 15 studies (1,556 cases; 1,720 controls), Bhutta et al. (2002) calculated a mean FSIQ deficit among cases of 10.85 points, producing a total loss of 34,031,025 FSIQ points. The severity of preterm birth likely influences the magnitude of the deficit, but separate estimates for specific intervals of gestational age were not available. The estimate of 10.85 points is based on studies published between 1988 and 2001, and it is possible that the deficit among contemporary samples of children born preterm has been reduced by improvements in neonatal intensive care practices. In some cohorts born after 2000, FSIQ at age 5 years is approximately the expected mean of 100, even among infants born at 28 weeks of gestation and with birth weights of approximately 1,000 g (Lind et al. 2011).

Type 1 diabetes. The prevalence of type 1 diabetes in U.S. children is approximately 1 in 500 (~ 51,000 children). In a meta-analysis of 19 studies (1,393 cases; 751 controls), Gaudieri et al. (2008) estimated standardized effect sizes of –0.35 and –0.28 for two constructs, crystallized intelligence (i.e., ability to apply skills, knowledge, and experience) and fluid intelligence (i.e., ability to think logically to solve novel problems), respectively, in children with early-onset disease (diagnosis at < 7 years of age) compared with controls. The mean of these two, as a proxy for FSIQ, is –0.315, corresponding to 4.73 points. Among children with late-onset disease, the effect sizes for crystallized and fluid intelligence were –0.20 and –0.14, respectively, or a mean effect size of –0.17, corresponding to 2.55 points. If half of the cases are assumed to be early onset and half late onset, the total FSIQ loss is estimated to be 185,640 points.

Acute lymphocytic leukemia. The most common malignancy in children, acute lymphocytic leukemia, occurs with an annual incidence of 10 in 100,000, primarily in 2- to 5-year-olds, resulting in a total of 12,750 cases among children < 5 years of age in the United States. In a meta-analysis involving 28 studies of children treated with cranial radiation, intrathecal chemotherapy, or both, the average weighted standardized effect size for FSIQ was –0.71, corresponding to 10.65 points (Campbell et al. 2007). Therefore, the estimated FSIQ loss is 135,788 points.

Brain tumors. Brain tumors are the most common solid tumor diagnosis in children and the second leading cause of death by disease. The annual incidence is 2.35 in 100,000 children (Mulhern and Butler 2004), representing 599 cases yearly among U.S. children. In a meta-analysis of 32 studies involving 1,096 children, Robinson et al. (2010) reported a weighted standardized effect size of –0.83 for FSIQ, corresponding to a loss of 12.45 points. Therefore, the total FSIQ loss is 37,288 points. Significant heterogeneity of the effect sizes across studies was noted, most likely reflecting cohort differences in tumor subtype (e.g., medulloblastoma vs. astrocytoma) or differences in treatment protocols. Because of data limitations, however, tumor-specific estimates could not be made.

Duchenne muscular dystrophy. Duchenne muscular dystrophy is the most common form of muscular dystrophy, occurring in 2.9 in 10,000 male births (therefore, approximately 1.5 in 10,000 total births [Centers for Disease Control and Prevention (CDC) 2009a]. This corresponds to 3,825 cases in U.S. children 0–5 years of age. In a meta-analysis of 32 studies, which included 1,231 patients, Cotton et al. (2005) estimated a mean FSIQ of 82, representing a deficit of 18 points. Therefore, the total FSIQ loss is estimated to be 68,850 points.

*Neurodevelopmental disorders.* Autism spectrum disorders. The prevalence of autism spectrum disorders (ASDs) in the United States is approximately 1 in 110 (CDC 2009b), or 231,818 children in the 0- to 5-year age range. Although a meta-analysis of FSIQ scores of children with ASDs could not be found, Charman et al. (2011) conducted a population-based study in the United Kingdom that involved identifying each child in a population of 56,946 who had either a local diagnosis of an ASD or a “Statement of Educational Needs.” FSIQ was measured in the 156 children identified. The percentages of children in severity categories were as follows: 7.4% severe/profound (FSIQ < 35, or a loss of 65 points); 8.4% moderate (FSIQ 35–49, or an average loss of 58 points); 39.4% mild (FSIQ 50–69, or an average loss of 41 points); and 16.6% below average (FSIQ 70–84, or an average loss of 23 points). Another 25.4% were in the average range (85–114). Half of these children (12.7%) were assumed to have FSIQ scores between 85 and 99, with an average loss of 8 points. If these figures are applied to U.S. children, the estimated total FSIQ loss is 7,109,899 points.

Bipolar disorder. Based on the number of office visits that identify pediatric bipolar disorder as the reason (from the National Ambulatory Medical Care Survey of the National Center for Health Statistics, 1999–2003), Moreno et al. (2007) estimated the prevalence to be 6.67%. The weighted standardized effect size for FSIQ based on 10 studies included in a meta-analysis is –0.32, corresponding to a loss of 4.8 points (Joseph et al. 2008), producing an estimated total loss of 8,164,080 points.

Attention deficit hyperactivity disorder. Based on parent report (from the National Survey of Children’s Health, United States, 2007), 7.2% of children 4–17 years of age have a current diagnosis of attention deficit hyperactivity disorder (ADHD) (CDC 2010a). In a meta-analysis that included 137 comparisons of FSIQ among children with ADHD and controls (Frazier et al. 2004), the weighted standardized effect size was –0.61 or 9.15 points, producing an estimated total loss of 16,799,400 points.

*Postnatal events.* Traumatic brain injury. The number of cases of traumatic brain injury (TBI) is estimated to be 475,000 annually among children 0–14 years of age, with the highest incidence among children 0–4 years of age (Langlois et al. 2005). An assumption that 40% of TBI events occur in children 0–5 years of age results in an estimate of ~ 950,000 cases in the United States. In a meta-analysis, Babikian and Asarnow (2009) provided estimates of the mean FSIQ deficits associated with TBI of differing severity, defined by Glasgow Coma Scale score (mild: 13–15; moderate: 9–12; severe: 3–8). Because recovery does occur, mostly in the 24 months after injury, the estimated FSIQ deficits present 24 months postinjury were used. The mean deficits associated with mild, moderate, and severe TBI were 4.47, 8.99, and 16.59 points, respectively. Narayan et al. (2002) reported that 80% of TBIs are mild, 10% moderate, and 10% severe. Therefore, the FSIQ loss associated with mild TBI is 3,397,200 points; moderate TBI, 854,050 points; and severe TBI, 1,576,050. The total estimated loss is therefore 5,827,300 points.

*Socioeconomic, nutritional, and psychosocial risks.* Nonorganic failure to thrive. Some children fail to grow at the expected rate because of child abuse, neglect, parental mental disorder, and the like. Various criteria are used to identify cases, including weight below the 5th or 3rd percentile, a decrease of two major percentile lines on a growth chart, and < 80% expected weight. In a meta-analysis of studies in which cases were identified from primary care settings, Corbett and Drewett (2004) reported a difference of 4.2 points in the FSIQ scores of cases (*n* = 502) and controls (*n* = 523). The various definitions used imply different prevalences, but if as many as 5% of children are assumed to be cases, the total estimated loss is 5,355,000 points.

Iron deficiency. In the National Health and Nutrition Examination Survey (NHANES) 1999–2000, the prevalence of iron deficiency, defined as an abnormal value on two or more indicators (serum ferritin, transferring saturation, free erythrocyte protoporphyrin) was 7% in 1- to 2-year-olds and 5% in 3- to 5-year-olds (CDC 2002). In a meta-analysis of four supplementation trials (Sachdev et al. 2005), the weighted standardized effect size was 0.41, corresponding to an increase of 6.15 points in the FSIQ scores of iron-deficient children who received supplementation. Interpreting this value as an estimate of the loss among children who do not receive supplementation and assuming a prevalence of 6%, the estimated FSIQ loss in U.S. children from iron deficiency is 9,409,500 points.

*Environmental chemical exposures.* Methylmercury. Using NHANES 1999–2000 data, McDowell et al. (2004) reported the distribution of hair mercury levels in U.S. women of childbearing age (16–49 years). Levels for the 10th, 25th, 50th, 75th, 90th, and 95th percentiles were 0.04, 0.09, 0.19, 0.42, 1.11, and 1.73 µg/g, respectively. Axelrad et al. (2007) conducted a dose–response analysis that integrated the results of three major epidemiological studies (New Zealand, Faroe Islands, Seychelles Islands), identifying a regression coefficient of –0.18 child FSIQ points per microgram per gram increase in maternal hair during pregnancy. This coefficient was derived on the basis of maternal hair mercury levels considerably greater than those of U.S. women (means in the Seychelles Islands and Faroe Islands cohorts were 6.8 µg/g and 4.3 µg/g, respectively). To estimate FSIQ loss, it was assumed that the coefficient applies to hair mercury levels > 1.11 µg/g (90th percentile), approximately the value on which the methylmercury reference dose is based (Rice et al. 2003). A concentration of 1.73 µg/g (95th percentile) was assumed to be the midpoint hair mercury level of the 10% of U.S. women with a level > 1.11 µg/g. The estimated total FSIQ loss is therefore 284,580 points.

Some studies have reported adverse neurodevelopmental outcomes at maternal hair levels lower than those represented in the analyses by Axelrad et al. (e.g., Oken et al. 2008). If the slope of –0.18 FSIQ points per microgram per gram is assumed to hold over the full range of maternal hair mercury levels in U.S. women, the total loss would be 1,875,017 points.

Recent studies, as well as reanalyses of older studies, suggest that neurotoxicity of methylmercury is underestimated if account is not taken of the fact that fish consumption also provides exposure to beneficial nutrients (Budtz-Jorgensen et al. 2007; Lynch et al. 2011; Oken et al. 2008). Because this was not done in the analyses by Axelrad et al. (2007), the coefficient might underestimate the true slope and thus result in an underestimate of the total FSIQ loss.

Although NHANES data are nationally representative, concern has been expressed that the relatively small sample size (*n* = 1,726) resulted in inadequate sampling of subgroups at particular risk, such as the families of subsistence and sport fishermen (Knobeloch et al. 2007) and certain ethnic groups in which fish is a particularly prominent part of the diet (Hightower et al. 2006) and among whom awareness of fish advisories is low (Knobeloch et al. 2005). If consumers of a diet high in fish are indeed underrepresented in NHANES, the FSIQ loss calculated on the basis of the distribution of hair mercury levels in that survey would be an underestimate. When exposure of women of childbearing age is concerned, it is also important to consider whether the body burden for a chemical increases with age (e.g., polychlorinated biphenyls and, to a lesser extent, methylmercury). If burden is positively associated with age, risk will be overestimated unless age-specific natality rates are taken into account (Axelrad and Cohen 2011).

Organophosphate pesticides. In NHANES 1999–2004, the 5th, 10th, 25th, 50th, 75th, 90th, and 95th percentiles of the distribution of total urinary dialkyl phosphate (DAP) metabolites in pregnant women were 7.3, 9.3, 27.0, 65.0, 151.7, 237.2, and 483.5 nmol/L, respectively (CDC 2011b). A meta-analysis that provides the dose–effect relationship between total DAP metabolites and children’s FSIQ is not available, but Bouchard et al. (2011) and Engel et al. (2011) both reported on the association between total urinary DAP metabolites in pregnant women and childhood FSIQ. In the cohort studied by Engel et al. (2007), the median total urinary DAP metabolite concentration was 82 nmol/L (interquartile range of 35–195). In the cohort studied by Bouchard et al. (2011), the range was roughly 50–500 nmol/L. The ranges observed in these studies are therefore reasonable approximations of the upper half of the distribution of urinary DAP concentrations of U.S. pregnant women. Bouchard et al. (2011) reported that a 10-fold increase in total urinary DAP was associated with a loss of 5.6 FSIQ points [95% confidence interval (CI): –9.0, –2.2; *n* = 297], whereas Engel et al. (2011) reported that a 10-fold increase was associated with a loss of 1.39 points (95% CI: –4.5, 1.77; *n* = 140). Weighting the effect estimates by sample size produces an expected loss of 4.25 FSIQ points for a 10-fold increase in urinary DAP over this range, or a slope of –0.01 point/nmol/L. The studies do not provide information about the dose–effect relationship < 50 nmol/L, so this slope was applied only to the 50% of U.S. children with DAP levels > 65.0 nmol/L (the median). The studies also do not provide information about the relationship > 500 nmol/L, so the FSIQ loss for the 5% of U.S. children with a level > 483.5 nmol/L was assumed to be 4.25 points. Combining the slope and the distribution of total urinary DAP levels produces a total estimated loss of 16,899,488 points. If levels < 50th percentile are associated with FSIQ loss, the total would be greater.

Lead. In NHANES 2005–2006, the 50th, 75th, 90th, 95th, and 98th percentiles of the blood lead distribution for U.S. children 1–5 years of age were 1.43, 2.10, 2.98, and 3.80 µg/dL, respectively (CDC 2011a). The 98th percentile was not reported, but in NHANES 2003–2004, it was 10.0 µg/dL (CDC 2009c). Based on the percentage declines observed between NHANES 2003–3004 and 2005–2006 in the 90th and 95th percentiles (23.6% and 25.5%, respectively), I estimated that the 98th percentile in NHANES 2005–2006 was 7.5 µg/dL. Estimates of the dose–effect relationship relating blood lead level to FSIQ in children were taken from the pooled analysis of Lanphear et al. (2005). This analysis, involving > 1,300 children who participated in seven international prospective studies, identified a supralinear relationship over the range of 2.4–30 µg/dL. The best fits over narrower ranges were linear, however. An increase in blood lead level from 2.4 to 10 µg/dL was associated with a decrement of 3.9 FSIQ points (i.e., a slope of –0.51 points/µg/dL). This slope was also assumed to apply to blood lead levels between 0 and 2.4 µg/dL, as recent risk assessments have concluded that it is not possible to identify a blood lead level below which no adverse impact can be discerned [European Food Safety Authority 2010; Joint FAO/WHO (Food and Agriculture Organization/World Health Organization) Expert Committee on Food Additives 2010]. In the pooled analysis, an increase in blood lead from 10 to 20 µg/dL was associated with an additional decrement of 1.9 FSIQ points. A blood lead level of 15 µg/dL was assumed for the 2% of children with a blood lead level above the 98th percentile and an average loss of 6.1 points (5.1 points for the range of 0–10 µg/dL plus one additional point for the increase > 10 µg/dL). For children with blood lead levels < 10 µg/dL, the midpoint blood lead level for each interval was multiplied by –0.51 and the product then multiplied by the number of children in the interval. For children with levels > 10 µg/dL, the assumed midpoint of 15 µg/dL was multiplied by 6.1 points. The total estimated FSIQ loss is therefore 22,947,450 points (Table 1). An uncertainty analysis was conducted using the lower and upper bounds of the 95% CIs for the slopes estimated in the pooled analysis. Assuming the lower and upper bounds yields an estimated total FSIQ loss of 14,185,905 and 31,277,154 points, respectively.

## Discussion

Based on the estimated number of FSIQ points lost, the population burdens associated with environmental chemical exposures of children are surprisingly large—in some cases larger than those estimated for major medical conditions and events (Table 2). This is attributable not so much to the magnitude of the effect sizes associated with chemicals, but to the prevalence of exposures associated with adverse impacts. For example, in the case of lead, because of the absence of a threshold, every child contributes at least a little to population morbidity, and the cumulative impact is substantial, even if the lower bound of the CI for the effect size is used. Furthermore, most of the total morbidity is contributed by children with blood lead levels < 10 µg/dL rather than by children with levels > 10 µg/dL, the current CDC level of concern (Table 1). This illustrates the principle, applied in evaluating the impart of radiation exposure, that “it is the total dose falling on the whole population which determines the burden of health effects” (Rose 1991), and that “a large number of people at a small risk may give rise to more cases of disease than the small number who are at a high risk” (Rose 1985). This led Rose (1981) to argue in favor of supplementing individually based interventions, which target those at high risk, with population-based interventions designed to shift the population distribution of a health index in the direction of lower risk.

**Table 2:** Estimated FSIQ point losses associated with different risk factors in a population of 25.5 million children.

Risk factor	Total no. of FSIQ points lost
Medical conditions	
Congenital heart disease	104,805
Preterm birth	34,031,025
Type 1 diabetes	185,640
Acute lymphocytic leukemia	135,788
Brain tumors	37,288
Duchenne muscular dystrophy	68,850
Neurodevelopmental disorders	
ASDs	7,109,899
Pediatric bipolar disorder	8,164,080
ADHD	16,799,400
Postnatal traumatic brain injury	5,827,300
Socioeconomic, nutritional, psychosocial factors	
Nonorganic failure to thrive	5,355,000
Iron deficiency	9,409,500
Environmental chemical exposures	
Methylmercury	284,580
Organophosphate pesticides	16,899,488
Lead	22,947,450

Another illustration of the important distinction between individual and population risk is provided by the epidemiology of Down syndrome. As individuals, older women are at substantially greater risk than younger women of delivering a child with Down syndrome, as the relative risk associated with a maternal age > 44 years is approximately 50 compared with women < 30 years. The birth rate among younger women is so much higher, however, that 50% of children with Down syndrome are born to women < 30 years of age. Despite its large relative risk, maternal age has a modest attributable fraction, and much of the population burden is contributed by women who, individually, are at low risk. Although a factor that increases the odds ratio of a serious disease by 10-fold is clearly important for an affected individual, if it only increases the number of cases from 0.1 in 1,000,000 to 1 in 1,000,000, its import for population health is modest.

The notion that individuals with values on a health index that are not sufficiently extreme to meet diagnostic criteria may contribute substantially to population burden has been widely discussed in the context of chronic disease epidemiology. Applying categorical disease labels to individuals is necessary for clinical management and resource allocation. However, for many diseases that are defined using cutoff values of continuously distributed measurements (e.g., hypertension, diabetes, depression), the important question, as Rose (1993) noted, is not whether an individual “has” the disease but “how much of it” one has. Quoting Pickering, Rose (1993) lamented that “medicine in its present state can count up to two but not beyond,” tending to regard disease and health as nonoverlapping states. Goldberg (2000) observed that in insisting on categorical diagnoses, “rather than carving nature at the joints, we appear to be merely drawing lines in the fog.”

The strategies used to estimate the population health burden associated with chemicals have generally reflected a disease-oriented approach rather than a population-oriented approach. It is frequently noted that a modest shift in the mean IQ score in a population will be accompanied by a substantial increase in the percentage of individuals with extremely low scores. For example, Needleman et al. (1982) showed that the median verbal IQ scores of children with high and low dentine lead levels differed by only 5 or 6 points, but scores < 80 were three times more frequent in the high-lead group. This difference is surely important, but the cumulative frequency distributions of the two groups differed throughout the entire range and, in fact, never intersected. Focusing solely on the difference between the distributions in the extreme left tail fails to acknowledge that the differences over the rest of the range of verbal IQ scores are also germane to population burden.

A disease-oriented approach underlies the protocol used by the WHO to estimate the global burden of disease, in that it is only health states associated with an *International Classification of Diseases* (ICD) code that contribute to burden (WHO 2009). Fewtrell et al. (2003) used this protocol to estimate the burden attributable to lead. With regard to neurodevelopment, it was only children whose FSIQ score would be expected to be reduced to ≤ 70 (ICD category: mild mental retardation) as a result of lead exposure who contributed to the burden estimate. That a substantial fraction of the overall FSIQ loss in the calculations reported in this review is contributed by children with blood lead levels at the lower end of the exposure distribution, among whom few would be expected to meet criteria for mild mental retardation, indicates that a disease-oriented approach likely underestimates the contribution of lead to neurodevelopmental morbidity.

The population approach advocated here is consistent with the strategy economists use to assign monetary value to changes in FSIQ, which does not focus solely on the extreme low tail of the distribution. In estimating the economic benefits of reducing children’s lead exposure, Grosse et al. (2002) estimated that each FSIQ point lost reduces future work productivity by 1.76–2.38%, regardless of an individual’s initial FSIQ, a monetary loss placed by Gould (2009) at $17,815 in discounted lifetime earnings.

The important risk factors for FSIQ loss likely vary across cultural and regional settings. Although the intellectual burden associated with undernutrition, infectious diseases, and parasitic diseases is far greater among children in developing than in developed countries (Walker et al. 2007), exposures to environmental chemicals are also greater. In 2000, < 10% of children worldwide had a blood lead level > 20 µg/dL, but 99% of them lived in developing countries (Fewtrell et al. 2003), where tragic episodes of mass fatalities continue to occur (CDC 2010b).

The approach described in this review can be used to carry out a range-finding analysis of newly identified exposures and to prioritize research needs. For example, concern has been raised about the neurodevelopmental effects of children’s exposures to bisphenol A (Braun and Hauser 2011), polybrominated diphenyl ethers (Herbstman et al. 2010), and phthalates (Engel et al. 2010), but meta-analyses of these exposures have not been published. Keeping in mind that the effect estimates reported in initial studies of a risk factor tend to be larger than those reported in later studies (Ioannidis 2005), one can nevertheless estimate the total FSIQ loss using the limited data available to get a preliminary sense of how important an exposure might be on a population basis.

Finally, the approach can be helpful in evaluating the impact of interventions to reduce population exposures. Applying it to the blood lead level data collected in NHANES II (1976–1980) (Mahaffey et al. 1982) yields an estimated FSIQ loss of 119,990,842 points for that cohort of children, suggesting that measures taken in recent decades to reduce blood lead levels of children have produced a substantial reduction in neurodevelopmental morbidity. The approach could also be used to compare the anticipated benefits of alternative regulatory initiatives based on the expected impact of an initiative on a biomarker distribution.

## Limitations

*Use of FSIQ as the basis for calculations.* FSIQ was chosen as the end point for comparing risk factors, but this might not be the outcome that captures the most important impact of a risk factor. For example, among children with a TBI, deficits in visual-perceptual function are more prominent than deficits in FSIQ (Babikian and Asarnow 2009), and verbal memory and executive function are typically more impaired than FSIQ in children with bipolar disorder (Joseph et al. 2008). Because FSIQ averages performance over multiple domains, it is a poor metric for expressing the impact of a risk factor that selectively impairs certain domains, as has been suggested for methylmercury (Grandjean et al. 2006). Some risk factors, such as environmental tobacco smoke, might have their greatest impact on a child’s risk of behavioral disorders such as ADHD (Froehlich et al. 2009; Lindberg et al. 2010). Therefore, for other end points, the relative importance of risk factors might differ from what was observed for FSIQ.

In some studies, the FSIQ scores of children with a risk factor were compared with the scores of children in the standardization sample of the test used, whereas in others it was compared with the scores of a control group. Each strategy has advantages. Use of a control group can provide better control for confounding when studying a risk factor that tends to occur more often in particular subgroups of children (e.g., sickle cell disease). However, if the control group differs from the exposed group on important predictors of neurodevelopment and if adjustment for such factors is inadequate, the effect size derived could over- or underestimate the true value, depending on the circumstances.

*Data used to support the calculations.* The calculations carried out for different risk factors varied in terms of the quantity of data available (i.e., the number of studies, the number of children contributing data), resulting in differences in the weight and strength of the evidence that a risk factor is causally related to children’s FSIQ. For some risk factors, such as fetal alcohol syndrome/alcohol-related neurodevelopmental disabilities and environmental tobacco smoke, a published meta-analysis could not be found.. For other risk factors, the available meta-analyses involved ecologic comparisons not suitable for calculating FSIQ loss. For fluorosis (Tang et al. 2008) and arsenicosis (Dong and Su 2009), for example, meta-analyses reported only the mean FSIQ deficits of children in areas with endemic disease compared with control areas.

Although relying on a meta-analysis reduces the problems associated with selecting a single study as the basis for calculations, such an analysis might not be accurate if it included studies that suffer from biases. Here I relied on the subject-matter experts who conducted the analyses to select the data on which to base the effect estimates. In some analyses, the effect estimates reported showed significant heterogeneity. This might reflect the presence of effect modification by characteristics that varied across studies (Bellinger 2009) or to methodological factors such as differences in the amount of exposure measurement error, which usually biases estimation toward the null (Grandjean et al. 2004). It could also be attributable to the presence of residual confounding in some of the studies included. Historically, confounding has been of prime importance, and the focus of most contention, in studies of environmental chemical exposures, because of the frequent regulatory and economic implications of the causal inferences drawn. Equivalent attention might not be paid to such issues in studies of other risk factors and, to the extent that confounders have not been identified and adjusted for in the individual studies, the overall effect estimates derived in meta-analyses might be inaccurate.

For iron deficiency, the results of intervention trials provided the effect estimate. Even if studies suggest that supplementation improves cognitive outcomes, the effect size might underestimate the FSIQ loss if supplementation does not fully reverse the impact of iron deficiency. In a meta-analysis of Chinese studies, the FSIQ deficit of children living in iodine-deficient areas was 12.45 points compared with children from iodine-sufficient areas (Qian et al. 2005). Children from iodine-deficient areas in which women received iodine supplementation during pregnancy recovered only 8.7 points, however. Prospective observational studies that do not involve supplementation would provide purer estimates of the impact of nutrient deficiencies, although ethical considerations might prevent such studies

For some risk factors (e.g., iron deficiency, methylmercury), the effect estimates were derived from studies conducted on non-U.S. samples. Important regional differences in the distributions of potential effect modifiers could reduce the appropriateness of extrapolating the effect estimates to U.S. children. For example, the impact of a micronutrient deficiency might be greater among children with comorbid health conditions, and the effect estimate derived on the basis of such a sample would overestimate the impact of the deficiency among children who lack the comorbidities. Also, the effect estimates derived in some meta-analyses (e.g., of methylmercury) were derived on the basis of exposures that differ substantially from those of U.S. children, potentially compromising their usefulness.

*Conceptual issues.* Some risk factors for which calculations were made are likely to lie on the same causal pathway as other risk factors. For example, because increased exposure to air pollutants has been associated with preterm birth, some portion of the burden calculated for preterm birth might represent an indirect effect of such exposures (Llop et al. 2010). In some cases, it might be possible to disentangle the contributions of different risk factors. For instance, Braun et al. (2006) estimated that one in five ADHD cases can be attributed to children with a blood lead level > 2 µg/dL. The WHO Global Burden of Disease methodology addresses this general problem by establishing disease “envelopes” (WHO 2009). For example, the total number of known deaths from hepatocellular carcinoma establishes an upper bound for the number of cases that can be attributed to its various causes (e.g., hepatitis B, hepatitis C, alcoholism, aflatoxin exposure). If the number of deaths attributed to all causes exceeds this total, double counting has likely occurred. Because FSIQ loss is not a reported disease, however, such bounding cannot be used to constrain the calculations.

Finally, it is likely that risk factors interact with one another to influence FSIQ of children. For example, not only do children living in poverty tend to experience greater exposures to environmental chemicals than do children living in advantaged circumstances (Elliott et al. 2004), but increasing evidence also suggests that material hardship, increased stress, and lack of enrichment opportunities exacerbate chemical neurotoxicity (Weiss and Bellinger 2006). Because of limitations in the available data, the impact of such interactions could not be estimated.

## Conclusion

Any effort to compare the neurodevelopmental burden associated with different risk factors is limited by the data available and the assumptions required. It was possible to estimate the total loss of FSIQ points in the population of 0- to 5-year-old U.S. children for a variety of risk factors, including three environmental chemicals: methylmercury, organophosphate pesticides, and lead. Despite the limitations of the approach, it appears that when population impact is considered, the contributions of chemicals to FSIQ loss in children are substantial, in some cases exceeding those of other recognized risk factors for neurodevelopmental impairment in children. The primary reason for this is the relative ubiquity of exposure. As a community, we have not effectively communicated this point to risk assessors and other decision makers, despite the fact that a risk assessment that focuses solely on individual risk and fails to consider the problem in a public health context is potentially misleading.
